# Prognostic analysis of radical resection for intrahepatic cholangiocarcinoma: a retrospective cohort study

**DOI:** 10.18632/oncotarget.18465

**Published:** 2017-06-13

**Authors:** Qingqiang Ni, Weifeng Shen, Minfeng Zhang, Cheng Yang, Wenchang Cai, Mengchao Wu, Jiamei Yang

**Affiliations:** ^1^ Medical College of Soochow University, Suzhou, Jiangsu, China; ^2^ Department of Special Treatment and Liver Transplantation, Eastern Hepatobiliary Surgery Hospital, Second Military Medical University, Shanghai, China

**Keywords:** intrahepatic cholangiocarcinoma, radical resection, disease-free survival, overall survival, retrospective cohort study

## Abstract

The aim of this study was to investigate the relationship between the clinicopathological characteristics of intrahepatic cholangiocarcinoma (ICC) and both disease-free survival (DFS) and overall survival (OS) in intrahepatic cholangiocarcinoma (ICC) patients who underwent radical resection (R0). We retrospectively analyzed the clinicopathological characteristics of 319 patients who underwent radical resection of ICC between October 1999 and December 2003. The independent adverse prognostic factors that affected DFS after radical resection of ICC were as follows: maximum tumor diameter (HR = 1.330, *P* = 0.014), complicated bile duct stone (HR = 1.923, *P* = 0.013), macroscopic tumor thrombus (HR = 1.826, *P* = 0.009), and lymph node metastasis (Pathology N1) (HR = 2.330, *P* = 0.005) were independent adverse prognostic factors that affected the DFS after radical resection of ICC. The postoperative median DFS was 6 months. The independent adverse prognostic factors that affected OS after radical resection of ICC were as follows: maximum tumor diameter (HR = 1.326, *P* = 0.014), complicated bile duct stone (HR = 2.349, *P* = 0.001), and lymph node metastasis (Pathology N1) (HR = 2.420, *P* = 0.003). The postoperative median survival time was 22 months, the 3-year survival rate was 33.9%, and the 5-year survival rate was 23.2%. Macroscopic tumor thrombus (OR = 2.991, *P* = 0.004) was an independent risk factor for death within 1 year after radical resection.

## INTRODUCTION

Globally, primary liver carcinoma (PLC) is the fifth most common malignant tumor, resulting in 600,000 deaths each year [1]. Intrahepatic cholangiocarcinoma (ICC) is a carcinoma arising from the epithelial cells of the intrahepatic bile duct branches of the second-order or beyond. Although ICC is rare in clinical practice, its incidence and mortality rates have increased gradually in the past 30 years [2-7]. The incidence of ICC accounts for 10% of all cholangiocarcinomas and 3% of gastrointestinal carcinomas [8, 9].

ICC is caused by a variety of etiologies, such as hepatitis B, hepatitis C, AIDS, intrahepatic bile duct stones, liver cirrhosis, primary sclerosing cholangitis, parasitic infections, chemical carcinogens, obesity, type II diabetes, and nonalcoholic fatty liver disease [10]. With the continuous improvement of the quality of life in China, the incidences of excessive alcohol consumption, obesity, type II diabetes, and nonalcoholic fatty liver disease have increased. As early as 2006, the number of patients with chronic hepatitis B in China had reached 20 million [11]. The abovementioned factors may be responsible for the world’s highest incidence of ICC being reported in Asia, including in China [12]. The median survival time in ICC patients is as low as 2-6 months if only palliative treatment is involved. Moreover, the survival time is over 5 years in less than 10% of patients, regardless of the treatment regimen [3, 13, 14]. However, the 5-year survival rate after surgical resection ranges from 30-35% [15]. Although hepatectomy is the main treatment method for early ICC, only approximately one-third of ICC patients have an opportunity to undergo resection. The median survival time is only approximately 3 years, and the recurrence rate is as high as 50-60%; at the time of ICC diagnosis, the majority of patients are in the late stage and missed their opportunity to receive radical resection [16]. A high grade of malignancy, lower radical resection rate, and poor prognosis have resulted in a tremendous effect on ICC patients; therefore, this topic is worth further study.

With an increase in its aging population, China now has a high incidence of ICC, which deserves our full attention. Therefore, this retrospective cohort study was designed to determine the prognostic factors that can affect postoperative disease-free survival (DFS) and overall survival (OS) according to survival data and clinicopathological characteristics in the follow-up of 319 patients who underwent radical resection of ICC. This study aims to provide references for the clinical diagnosis and treatment of ICC.

## MATERIALS AND METHODS

### General data

This single-center, retrospective cohort study was approved by the ethical committee of the Shanghai Eastern Hepatobiliary Surgery Hospital. The study protocol conformed to the principles of the Declaration of Helsinki. The study subjects were selected from 319 patients who underwent radical resection (R0) of ICC from October 1999 to December 2003 in our hospital. Of these patients, 225 were male and 94 were female. Their average age was 52.70 ± 10.50 years (range: 22-81 years). A total of 109 patients (76 males and 33 females with a mean age of 53.44 ± 11.24 years) were included in the group with OS ≤ 1 year. The remaining 210 patients (149 males and 61 females with a mean age of 52.31 ± 10.10 years) were included in the group with OS > 1 year. The balance test showed that there were no significant differences (P > 0.05) in sex or age between the two groups. Thus, the data were compatible. All of the patients were diagnosed with ICC, and their diagnosis was confirmed by pathological examination. Patients with mixed HCC were excluded.

### Study methods

#### Clinical data collection

The clinical data of the patients were collected, including gender, age, tumor number, number of liver segments with tumors, perioperative blood transfusion, postoperative pathological type, the presence of liver cirrhosis, maximum tumor diameter, complicated bile duct stone, serum CA199 levels, macroscopic tumor thrombus, and tumor staging. According to their morphology, ICC can be graded as well, moderately and poorly differentiated adenocarcinoma. Well-differentiated ICCs include tubular adenocarcinoma with/without micropapillary structures; moderately differentiated ICCs include moderately distorted tubular glands with cribriform formations and/or a cord-like pattern; and poorly differentiated ICCs include distorted tubular or cord-like structures with marked cellular pleomorphism (WHO classification of tumors of the digestive system) Complicated bile duct stones were defined as the presence of calculi within the intrahepatic biliary tree proximal to the confluence of the left and right hepatic ducts. Tumor staging in all of the patients was determined according to the UICC TNM classification of malignant tumors, 7th edition.

#### Definition of radical resection

Radical hepatectomy primarily includes regular hepatectomy and hemihepatectomy, irregular hepatectomy, extended hepatectomy, and concomitant removal of bile duct stone or regional lymphadenectomy. The following criteria should be met: (1) all tumors are removed without residual tumor; (2) negative margins are confirmed by histological examination; (3) there is no major vascular invasion; and (4) preoperative elevated CA199 levels return to normal within 2 months after surgery. The preoperative assessment of liver function was based on the Child-Pugh scoring system. The surgical method and resection region were determined according to the tumor size and distribution and the hepatic functional reserve. Whether extended hepatectomy was performed was based on an evaluation of the volume of the remaining liver based on the results of a preoperative computed tomography (CT) scan or magnetic resonance imaging (MRI). The clamp crushing and finger fracture methods were used for anatomical liver resection at room temperature, with intermittent use of Pringle’s maneuver. The distance between the tumor and cut edge was 1-1.5 cm. The detection of regional lymph node metastases was based on CT/MRI/PET-CT. If regional lymph node metastases are suspected, regional lymphadenectomy should be considered, including complete excision of soft tissue and lymph nodes at the hepatic hilum, common hepatic artery, hepatoduodenal ligament, portal vein and posterior to the pancreatic head stations. A definitive diagnosis was made by pathologic evidence.

#### Follow-up

Follow-up was performed in all of the patients by telephone communication or outpatient visits. The observed outcomes included DFS and OS. The initial event to assess DFS was radical hepatectomy, and the endpoint was the time of tumor recurrence or the occurrence of a censored event. The initial event to assess OS was radical hepatectomy, and the endpoint event was the death of the patient or the occurrence of a censored event. Censored events included the following: (1) loss of patients during follow-up; and (2) no evidence of tumor recurrence or the patient was still alive at the end of the follow-up. Recurrence was diagnosed based on imaging of B-mode ultrasound, CT scan, and MRI, or on confirmation of ICC recurrence by biopsy and pathological examinations. The starting time of follow-up was the time point when radical hepatectomy was performed, and the endpoint was May 15, 2016. Loss of follow-up was defined as the occurrence of the following events: the patient had missed their outpatient visits for 6 months; the patient could not be reached by phone; or the patient was not hospitalized for 6 months. In this study, 118 cases were lost during the follow-up, and 201 cases completed the follow-up. The follow-up rate was 63.01% (201/319). The follow-up duration ranged between 1 and 177 months, with a median follow-up duration of 13 months.

#### Statistical analysis

The statistical analysis was performed with SPSS 17.0 software. The chi-square test was used for the count data, and the rank sum test was used for the ranked data. A multivariate analysis for shorter OS was performed using a binary logistic regression. The survival rate was calculated using the Kaplan-Meier method. The survival rates were compared using the log-rank method. A Cox regression model was used to analyze the prognostic factors. A P value of less than 0.05 was considered statistically significant.

## RESULTS

### Clinicopathological characteristics of the 319 patients who underwent radical resection of ICC

Of the 319 ICC patients, 225 patients were male, and 94 patients were female, with an average age of 52.70 ± 10.50 years (range: 22-81 years). A statistical description of the clinicopathological characteristics for these patients is shown in Table 1.

**Table 1:** Clinicopathological characteristics of 319 patients who underwent radical resection of ICC

	Number of cases	Total number of cases	Percentage (%)
Gender		319	
male	225		70.5
female	94		29.5
Age		319	
> 55 years	126		39.5
≤ 55 years	193		60.5
Single tumor		319	
yes	229		71.8
no	90		28.2
Tumor distribution		319	
> 2 hepatic segments	116		36.4
≤ 2 hepatic segments	203		63.6
Perioperative blood transfusion		319	
yes	137		42.9
no	182		57.1
Pathological type		291	
Well differentiated	4		1.4
Moderately differentiated	113		38.8
Poorly differentiated	174		59.8
Evidence of liver cirrhosis		319	
yes	123		61.4
no	196		38.6
Maximum tumor diameter (cm)		319	
≤5	127		39.8
>5, ≤10	139		43.6
>10	53		16.6
Complicated bile duct stone		318	
yes	30		9.4
no	288		90.6
Serum CA199 (U/ml)		313	
>37	174		55.6
≤37	139		44.4
Macroscopic tumor thrombus		319	
yes	39		12.2
no	280		87.8
Pathology T		319	
T1	10		3.1
T2	130		40.8
T3	86		27
T4	93		29.2
Pathology N		319	
N0	293		91.8
N1	26		8.2
Pathology M		319	
M0	316		99.1
M1	3		0.9
Pathology stage		319	
I	10		3.1
II	119		37.3
III	97		30.4
IV	93		29.2

### Analysis of the impact factor for DFS after radical resection

**Figure 1:**
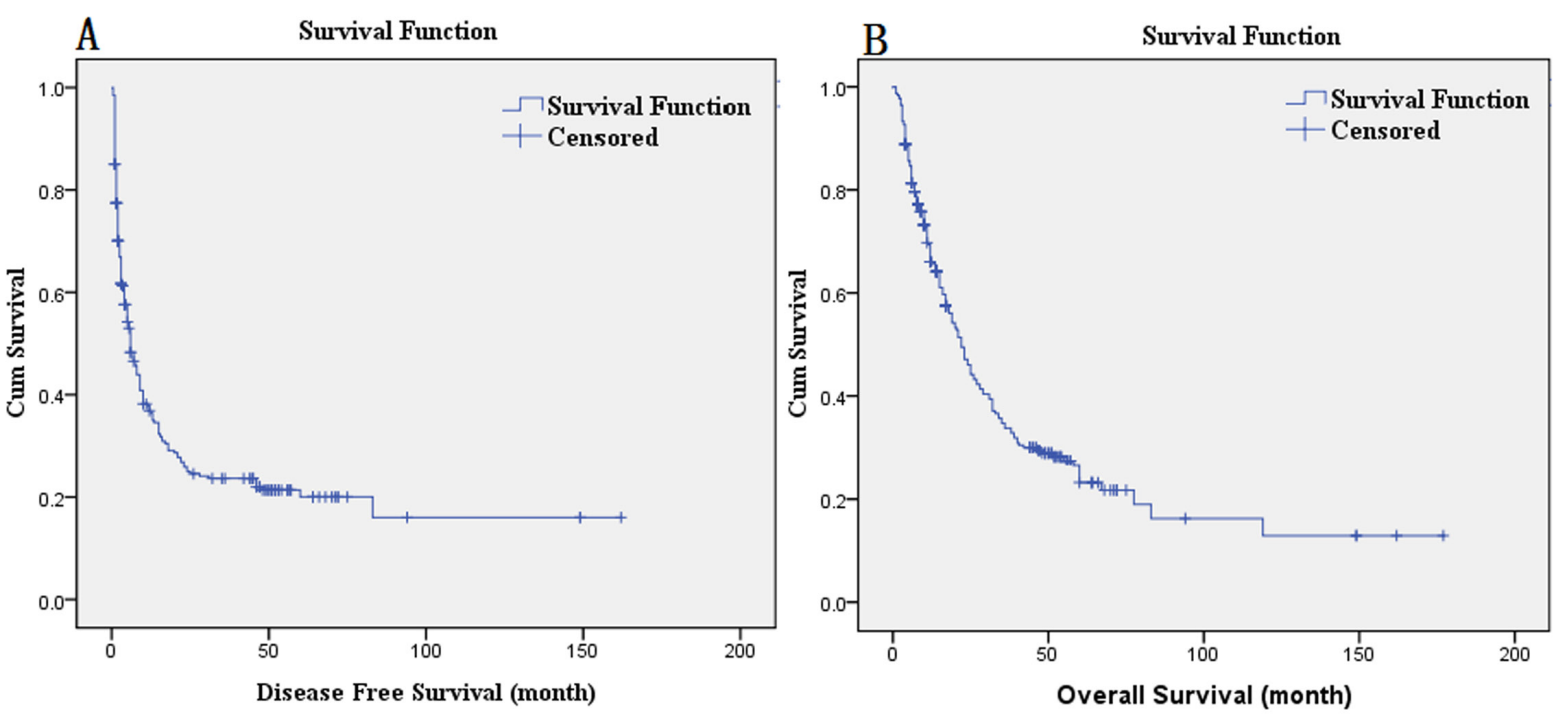
**A.** DFS curves; **B.** OS curves.

The univariate Cox analysis showed that the number of tumors, tumor distribution, perioperative blood transfusion, maximum tumor diameter, complicated bile duct stone, serum CA199 levels (U/ml), macroscopic tumor thrombus, Pathology T, Pathology N, and pathology stage were the prognostic factors that affected the DFS in patients who underwent radical hepatectomy for ICC (*P* < 0.05). The significant prognostic factors determined by the univariate analysis were included in a multivariate Cox analysis, which showed that maximum tumor diameter (HR = 1.330, *P* = 0.014), complicated bile duct stone (HR = 1.923, *P* = 0.013), macroscopic tumor thrombus (HR = 1.826, *P* = 0.009), and Pathology N (HR = 2.330, *P* = 0.005) were independent adverse prognostic factors that affected the DFS of patients who underwent radical hepatectomy for ICC (Table 2). The median DFS was 6 months; the DFS curves are shown in Figure 1A.

**Table 2:** Analysis of impact on DFS after radical resection

Clinicopathological characteristics	Number of cases	Univariate analysis			Multivariate analysis		
		**HR**	***P***	**95% CI**	**HR**	***P***	**95% CI**
Gender		0.917	0.576	(0.676, 1.244)			
male	225						
female	94						
Age		1.264	0.106	(0.951, 1.680)			
> 55 years	126						
≤ 55 years	193						
Single tumor		1.621	0.002	(1.193, 2.202)	1.022	0.948	(0.538, 1.940)
yes	229						
no	90						
Tumor distribution		1.222	0.002	(1.079, 1.348)	1.511	0.226	(0.775, 2.945)
> 2 hepatic segments	116						
≤ 2 hepatic segments	203						
Perioperative blood transfusion		1.410	0.019	(1.059, 1.877)	0.998	0.993	(0.698, 1.427)
yes	137						
no	182						
Pathological type		1.082	0.593	(0.810, 1.445)			
Well differentiated	4						
Moderately differentiated	113						
Poorly differentiated	174						
Evidence of liver cirrhosis		0.913	0.535	(0.683, 1.219)			
yes	123						
no	196						
Maximum tumor diameter (cm)		1.447	0.000	(1.189, 1.761)	1.330	0.014	(1.060, 1.669)
≤5	127						
>5, ≤10	139						
>10	53						
Complicated bile duct stone		1.718	0.020	(1.089, 2.708)	1.923	0.013	(1.145, 3.227)
yes	30						
no	288						
Serum CA199 (U/ml)		1.456	0.011	(1.089, 1.946)	1.266	0.486	(0.652, 2.457)
>37	174						
≤37	139						
Macroscopic tumor thrombus		1.739	0.009	(1.146, 2.637)	1.826	0.009	(1.160, 2.873)
yes	39						
no	280						
Pathology T		1.363	0.000	(1.158, 1.604)	1.727	0.091	(0.917, 3.251)
T1	10						
T2	130						
T3	86						
T4	93						
Pathology N		2.156	0.005	(1.261, 3.686)	2.330	0.005	(1.297, 4.186)
N0	293						
N1	26						
Pathology M		1.245	0.759	(0.308, 5.028)			
M0	316						
M1	3						
Pathology stage		1.383	0.000	(1.172, 1.630)	0.986	0.972	(0.434, 2.239)
I	10						
II	119						
III	97						
IV	93						

### Impact factor analysis of OS after radical resection

**Figure 2:**
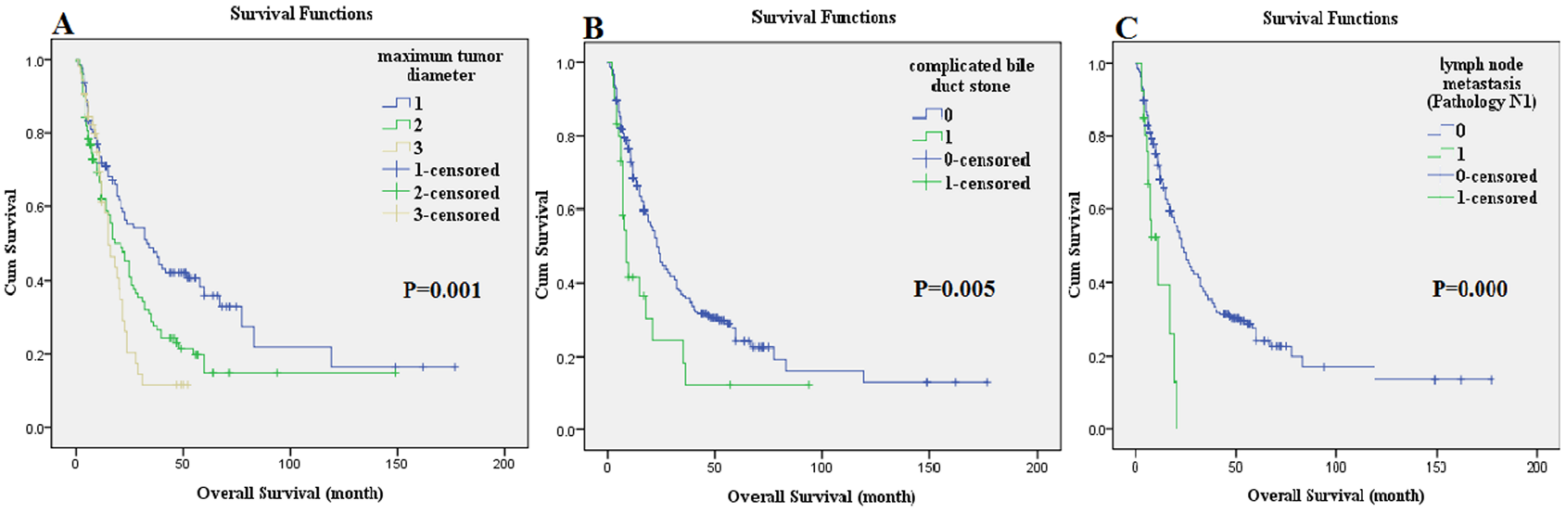
The survival comparison of independent prognostic factors using the log-rank method: maximum tumor diameter **A.**, complicated bile duct stone **B.**, and lymph node metastasis (Pathology N1) **C.**.

The univariate Cox analysis showed that the tumor distribution, maximum tumor diameter, complicated bile duct stone, serum CA199 level (U/ml), Pathology T, Pathology N, and pathology stage were the prognostic factors for OS in patients who underwent radical hepatectomy for ICC (*P* < 0.05). The significant prognostic factors determined by the univariate analysis were included in the multivariate Cox analysis, which showed that the maximum tumor diameter (HR = 1.326, *P* = 0.014), complicated bile duct stone (HR = 2.349, *P* = 0.001), and Pathology N (HR = 2.420, *P* = 0.003) were independent adverse prognostic factors that affected OS of patients who underwent radical hepatectomy for ICC (Table 3). The postoperative median survival time was 22 months; the 3-year survival rate was 33.9%; and the 5-year survival rate was 23.2%. The OS curves are shown in Figure 1B. The survival comparison by log-rank method of independent prognostic factors, such as maximum tumor diameter, complicated bile duct stone, and Pathology N, are shown in Figure 2.

**Table 3:** Analysis of impact on OS after radical resection

Clinicopathological characteristics	Number of cases	Univariate analysis			Multivariate analysis		
		**HR**	***P***	**95% CI**	**HR**	***P***	**95% CI**
Gender		0.915	0.566	(0.675, 1.240)			
male	225						
female	94						
Age		1.193	0.229	(0.895, 1.589)			
> 55 years	126						
≤ 55 years	193						
Single tumor		1.267	0.132	(0.932, 1.723)			
yes	229						
no	90						
Tumor distribution		1.710	0.000	(1.272, 2.299)	1.209	0.331	(0.825, 1.774)
> 2 hepatic segments	116						
≤ 2 hepatic segments	203						
Perioperative blood transfusion		1.274	0.097	(0.957, 1.697)			
yes	137						
no	182						
Pathological type		1.173	0.272	(0.882, 1.560)			
Well differentiated	4						
Moderately differentiated	113						
Poorly differentiated	174						
Evidence of liver cirrhosis		0.888	0.424	(0.662, 1.189)			
yes	123						
no	196						
Maximum tumor diameter (cm)		1.451	0.000	(1.190, 1.770)	1.326	0.014	(1.059, 1.660)
≤5	127						
>5, ≤10	139						
>10	53						
Complicated bile duct stone		1.874	0.007	(1.187, 2.960)	2.349	0.001	(1.397, 3.950)
yes	30						
no	288						
Serum CA199 (U/ml)		1.494	0.007	(1.115, 2.001)	1.178	0.309	(0.859, 1.617)
>37	174						
≤37	139						
Macroscopic tumor thrombus		1.422	0.109	(0.925, 2.187)			
yes	39						
no	280						
Pathology T		1.272	0.004	(1.080, 1.498)	0.636	1.248	(0.499, 3.125)
T1	10						
T2	130						
T3	86						
T4	93						
Pathology N		2.578	0.001	(1.491, 4.458)	2.420	0.003	(1.340, 4.372)
N1	26						
N0	293						
Pathology M		1.588	0.516	(0.393, 6.413)			
M0	316						
M1	3						
Pathology stage		1.299	0.002	(1.101, 1.532)	0.912	0.949	(0.375, 2.398)
I	10						
II	119						
III	97						
IV	93						

### Risk factor analysis for shorter OS (OS ≤ 1 year) after radical resection

We defined shorter OS as OS ≤ 1 year. A total of 109 patients (76 males and 33 females with a mean age of 53.44 ± 11.24 years) were included in the group with OS ≤ 1 year. The remaining 210 patients (149 males and 61 females with a mean age of 52.31 ± 10.10 years) were included in the group with OS > 1 year. The balance test showed that there was no significant difference (P > 0.05) in sex or age between the 2 groups. Thus, the data were compatible. The univariate analysis showed that the tumor number, tumor distribution, complicated bile duct stone, macroscopic tumor thrombus, and Pathology T may be relevant factors that result in a postoperative shorter OS (*P* < 0.05). The binary logistic multivariate analysis showed that macroscopic tumor thrombus may be an independent risk factor for a shorter OS in ICC patients after radical resection (OR = 2.991, *P* = 0.004) (Table 4).

**Table 4:** Analysis of risk on shorter OS (OS ≤ 1 year) after radical resection

Clinicopathological characteristics	Number of cases	Univariate analysis			Multivariate analysis		
		**Group with OS ≤ 1 year**	**Group with OS>1 year**	***P***	**OR**	***P***	**95% CI**
Gender				0.820			
male	225	76	149				
female	94	33	61				
Age				0.477			
> 55 years	126	46	80				
≤ 55 years	193	63	130				
Single tumor				0.031	0.748	0.568	(0.275, 2.029)
yes	229	70	159				
no	90	39	51				
Tumor distribution				0.011	1.661	0.57	(0.986, 2.799)
> 2 hepatic segments	116	50	66				
≤ 2 hepatic segments	203	59	144				
Perioperative blood transfusion				0.216			
yes	137	52	85				
no	182	57	125				
Pathological type				0.160			
Well differentiated	4	1	3				
Moderately differentiated	113	32	81				
Poorly differentiated	174	68	106				
Evidence of liver cirrhosis				0.995			
yes	123	42	81				
no	196	67	129				
Maximum tumor diameter (cm)				0.071			
≤5	127	109	210				
>5, ≤10	139						
>10	53						
Complicated bile duct stone				0.021	2.755	0.232	(1.149, 6.605)
yes	30	16	14				
no	288	93	195				
Serum CA199 (U/ml)				0.077			
>37	174	68	106				
≤37	139	41	98				
Macroscopic tumor thrombus				0.000	2.991	0.004	(1.429, 6.260)
yes	39	23	16				
no	280	86	194				
Pathology T				0.000	1.237	0.324	(0.810, 1.890)
T1	10	109	210				
T2	130						
T3	86						
T4	93						
Pathology N				0.362			
N0	293	109	210				
N1	26						
Pathology M				0.211			
M0	316	109	210				
M1	3						
Pathology stage				0.057			
I	10	109	210				
II	119						
III	97						
IV	93						

## DISCUSSION

The prognostic factors for survival in ICC patients have not been fully elucidated, and many factors and mechanisms remain under investigation. In this study, follow-up (duration: 1-177 months) for survival was performed in 319 patients who underwent radical hepatectomy of ICC between October 1999 and December 2003 in our hospital. The median follow-up duration was 13 months; 118 cases were lost during the follow-up. The possible prognostic factors related to ICC were analyzed, and the high risk factors for poor prognosis in ICC patients were investigated.

In this study, a multivariate Cox analysis showed that the maximum tumor diameter, complicated bile duct stone, and lymph node metastasis were independent adverse prognostic factors that affected the DFS and OS in ICC patients after radical hepatectomy. Macroscopic tumor thrombus is an independent adverse prognostic factor for DFS in ICC patients after radical hepatectomy. Whether the tumor diameter affects survival time after hepatectomy has been controversial. The 6th edition of the AJCC TNM staging system showed that the presence of an isolated lesion or multiple lesions with a diameter ≤ 5 cm indicate a good prognosis. However, the staging system proposed by Okabayashi and Nathan and the 7th edition of the AJCC TNM staging system suggested that tumor diameter is not an independent prognostic factor for postoperative survival time. More recently, there are data to show that size does play a role up to a maximum size of 7 cm and then plateaus. Yamasaki suggested that ICC patients with tumors > 2 cm in diameter had a relatively poor prognosis [17]. This study has demonstrated that maximum tumor diameter was an independent adverse prognostic factor for survival time after radical resection of ICC. When the maximum tumor diameter increased by one level, the risk of recurrence and death increased 1.330 and 1.326 times, respectively. A study by Nordenstedt showed that gallstones can increase the risk of intrahepatic and extrahepatic cholangiocarcinoma [18]. Mechanisms of intrahepatic bile duct stone formation remain unclear and may be associated with malnutrition, low economic status, and biliary tract infections. Intrahepatic bile duct stones are closely correlated with the incidence of ICC and therefore may be related to the recurrence of ICC after radical resection [19-21]. In this study, the multivariate analysis showed that complicated bile duct stones are an independent adverse prognostic factor that can affect DFS in patients after radical resection of ICC. The impact of lymph node metastasis on postoperative survival in ICC patients remains debatable. The effect of intraoperative lymphadenectomy during the resection of ICC on prognosis is not yet supported by sufficient data. A study by Shimada et al. showed that intraoperative lymphadenectomy does not improve the prognosis of ICC patients [22]. Studies by Morine et al. have shown that prophylactic lymphadenectomy is not necessary for ICC patients who do not display lymph node metastasis [23]. The study by Li et al. showed that ICC patients without evidence of lymph node metastasis and ICC patients with multiple tumors and lymph node metastasis did not benefit from lymphadenectomy [24]. A systematic review by Amini et al. showed that the 3- and 5-year survival rates were only 0.2 and 0%, respectively, in ICC patients and lymph node metastasis. Although there are not sufficient data to support intraoperative routine lymphadenectomy, lymphadenectomy is recommended during the resection of ICC because of the higher rate of lymph node metastasis in ICC patients [25]. Some studies have shown that lymph node metastasis is an independent prognostic factor for postoperative survival in ICC patients [26-28]. This study also confirmed that lymph node metastasis was an independent adverse prognostic factor that affects DFS in patients after radical resection of ICC. Terada et al. have reported one case of portal hypertension caused by extensive portal vein tumor thrombi; that patient died of liver function failure 50 days after hospitalization [29]. This study indicated that the macroscopic tumor thrombus was an independent adverse prognostic factor for DFS in patients after undergoing radical resection of ICC. The study by Farges et al. suggested that a satellite lesion was a prognostic factor for OS in patients after undergoing radical resection of ICC; however, these authors were not able to demonstrate that a satellite lesion was an independent prognostic factor for OS in ICC patients [30]. In this group of patients, the postoperative median survival time was 22 months, the 3-year survival rate was 33.9%, and the 5-year survival rate was 23.2%. The 5-year survival rate was higher than that reported by Lang et al., Nathan et al., Jan et al., Ohtsuka et al., and Weimann et al. [31-35].

In a comparison between the group with OS ≤ 1 year (shorter OS) and the group with OS > 1 year, the univariate analysis showed that multiple tumors, tumor distribution, complicated bile duct stone, macroscopic tumor thrombus, and lymph node metastasis were possible risk factors for the shorter OS. The studies by Michael et al. revealed that the following factors led to a shorter OS: age, large size of tumor, multiple tumors, lymph node metastasis, vascular invasion, and poorly differentiated tumors [36]. In this study, the univariate analysis indicated that multiple tumors and lymph node metastasis are risk factors for shorter OS. These results are consistent with the findings of Michael et al. In the present study, significant risk factors identified by the univariate analysis were further included in the binary logistic multivariate analysis, which confirmed that macroscopic tumor thrombus may be an independent risk factor for a shorter OS. Studies by Zou et al. have shown that ARID1A, PBRM1, and BAP1, which are tumor suppressor genes, played an important role in suppressing mutations and preventing the occurrence of ICC. The mutation of any single gene in the three wild-type genes can lead to a shorter survival time [37]. This study provided references for further investigating the risk factors of shorter OS after radical resection of ICC.

In summary, radical resection is the only method to potentially cure ICC. However, most patients miss their opportunity for radical surgery because of the late stage of the disease at the time of diagnosis. Even when radical resection is performed, the high recurrence rate and short survival time can negatively affect both the clinicians and ICC patients. In this study, the multi-year follow-up demonstrated that the maximum tumor diameter, complicated bile duct stone, macroscopic tumor thrombus, and lymph node metastasis are independent adverse prognostic factors that can affect survival time in patients who undergo radical resection of ICC. Moreover, risk factors for shorter OS were analyzed. The results showed that macroscopic tumor thrombus is possibly an independent risk factor for mortality within 1 year in patients who undergo radical resection of ICC. The results of this study are intended to guide clinicians in identifying patients with high risks and to give appropriate treatments and interventions as early as possible to improve the life quality and postoperative survival rate of patients.
